# MITA Promotes Macrophage Proinflammatory Polarization and Its circRNA-Related Regulatory Mechanism in Recurrent Miscarriage

**DOI:** 10.3390/ijms24119545

**Published:** 2023-05-31

**Authors:** Bowen Liu, Jun Liu, Yang Qiu, Jiao Chen, Jing Yang

**Affiliations:** 1 Hubei Clinic Research Center for Assisted Reproductive Technology and Embryonic Development, Reproductive Medical Center, Renmin Hospital of Wuhan University, Wuhan 430060, China; bowenliu@whu.edu.cn; 2 Wuhan Children’s Hospital (Wuhan Maternal and Child Healthcare Hospital), Tongji Medical College, Huazhong University of Science & Technology, Wuhan 430016, China; 2019103020032@whu.edu.cn; 3 State Key Laboratory of Virology, Wuhan Institute of Virology, Center for Biosafety Mega-Science, Chinese Academy of Sciences (CAS), Wuhan 430071, China; yangqiu@wh.iov.cn

**Keywords:** MITA, macrophage polarization, apoptosis, circKIAA0391, miR-512-5p, recurrent miscarriage

## Abstract

MITA (also called STING), a master regulator of DNA-mediated innate immune activation, is a potential therapeutic target for viral infection and virus-related diseases. The circRNA-mediated ceRNA network plays important roles in gene regulation and may contribute to many human diseases. However, the relationship between MITA and recurrent miscarriage (RM) and its circRNA-related regulatory mechanisms remain unclear. In this study, we validated that the decidual M1/M2 ratio was upregulated in RM patients, suggesting the vital roles of decidual macrophages in the pathogenesis of RM. We found that MITA was highly expressed in decidual macrophages of RM patients and validated that MITA could promote apoptosis and macrophage proinflammatory polarization in THP-1-derived macrophage (TDM) cells. Using circRNA sequencing and bioinformatic analysis, we screened out a novel circRNA (circKIAA0391) that is overexpressed in decidual macrophages of RM patients. Mechanistically, we found that circKIAA0391 could promote the apoptosis and proinflammatory polarization of TDM cells by sponging the miR-512-5p/MITA axis. This study provides a theoretical basis for further understanding the impact of MITA on macrophages and its circRNA-related regulatory mechanisms, which may have a crucial immunomodulatory function in the pathophysiology of RM.

## 1. Introduction

According to the European Society of Human Reproduction and Embryology (ESHRE) guidelines, RM is defined as the failure of two or more clinically recognized pregnancies before 20–24 weeks of gestation and includes embryonic and fetal losses [1]. From an immunological perspective, since the embryo is a semi-allograft with paternal alloantigens, a successful pregnancy requires the maternal immune system to tolerate the semi-allogeneic fetus. The maternal–fetal interface, a dynamic site between the uterine mucosa and the extra-embryonic tissues of the developing conceptus, encompasses multiple cellular interactions in an environment rich in cytokines and hormones [2]. Mounting evidence has demonstrated that disruption of maternal immune tolerance is one possible cause of RM. Previous studies have demonstrated that first-trimester human decidual leukocytes are primarily natural killer (NK) cells (~70%), macrophages (~20%), and T cells (10–20%), while dendritic cells (DCs), B cells, and natural killer T cells (NKT cells) are rare [3]. A number of cytokines contribute to monocyte development into macrophages, which are known as uterine tissue-resident macrophages. There is a link between macrophages and embryo implantation, placental formation, embryonic development, and delivery [4]. Together with our growing understanding of the roles and mechanisms of macrophages during pregnancy, current evidence suggests that macrophages are essential.

Mediator of IRF3 activation (MITA), also known as stimulator of interferon genes (STING), is a transmembrane protein that plays a critical role in virus-induced type I IFN signaling, as demonstrated by expression cloning. As we know, MITA was also found to localize to the outer membrane of mitochondria and to be associated with VISA, a mitochondrial protein that acts as an adaptor in virus-triggered signaling [5]. Besides mediating the activation of IRF3 and NF-KB pathways and inducing the expression of IFN and proinflammatory cytokines, MITA also participates in other signal pathways and pathophysiological processes, such as autophagy, apoptosis, cell ageing, and the activation of inflammatory bodies [6]. As a crucial mediator of inflammation during infection, cellular stress, and tissue injury, the cGAS-STING signaling pathway has emerged as a key pathway. Decout, A. et al. discuss the cGAS–STING signaling cascade and its association with degenerative, auto-inflammatory, and immune diseases [7]. STING signaling could also be used to suppress covalently closed circular DNA (cccDNA) replication of hepatitis B virus (HBV) and alleviate liver fibrosis caused by HBV-induced macrophage inflammasome activation by activating autophagic flux in chronic HBV mice [8]. MITA’s regulatory effect on macrophages in RM patients is still largely unknown due to the limited number of studies conducted.

Competition between endogenous RNAs (ceRNA) regulates the expression of each other by sharing miRNA response elements (MREs) [9,10]. In addition to being an alternative function for messenger RNAs, this hypothesis offers a unifying function for long non-coding RNAs, pseudogene transcripts, and circular RNAs [11]. An intrinsic and exonic sequence are included within circular RNAs (circRNAs) formed by head-to-tail splicing of exons in a transcript. By forming a 3′,5′-phosphodiester bond between the 3′ end of an exon and the 5′ end of the upstream exon or its own, back-splicing junctions are created. circRNAs are stable transcripts found in both normal and malignant cells, and they may be encoded in thousands of human genes. Numerous studies have suggested that circRNAs may serve as promising disease markers due to their conservative properties [12]. It has been demonstrated that circTP63 can compete with miR-873-3p, inhibiting miR-873-3p from downregulating FOXM1, which eventually facilitates cell cycle progression by increasing CENPA and CENPB [13]. It has been suggested by Yao et al. [14] that the epigenetic regulator PRMT5 inhibits CAMK2N1 transcription, which may be a potential therapeutic target for aggressive prostate cancer patients by acting on the circSPON2/miR-331-3p axis. It is not known whether circRNAs are responsible for RM in macrophages, and what their specific mechanisms are.

In the present study, we first found that MITA was significantly upregulated in RM patients and may be correlated to the pathology of RM. In order to further distinguish the regulatory network of MITA in macrophages, circRNA microarray profiling was conducted to construct the differential circRNA expression profiles in decidual macrophages and found that Hsa_circ_0003241 (also known as circKIAA0391) was significantly upregulated in RM patients. Further functional experiments demonstrated that circKIAA0391 could promote TDM cell apoptosis and proinflammatory polarization. Mechanistically, we further demonstrated that circKIAA0391 exerts its functions by absorbing the miR-512-5p/MITA axis in TDM cells, which may participate in the pathology of RM. Thus, circKIAA0391 might be a promising biomarker for the diagnosis and treatment of RM.

## 2. Results

### 2.1. MITA Is Highly Upregulated in Decidual Macrophages Derived from RM Patients and Is Related to RM

**Figure 1:**
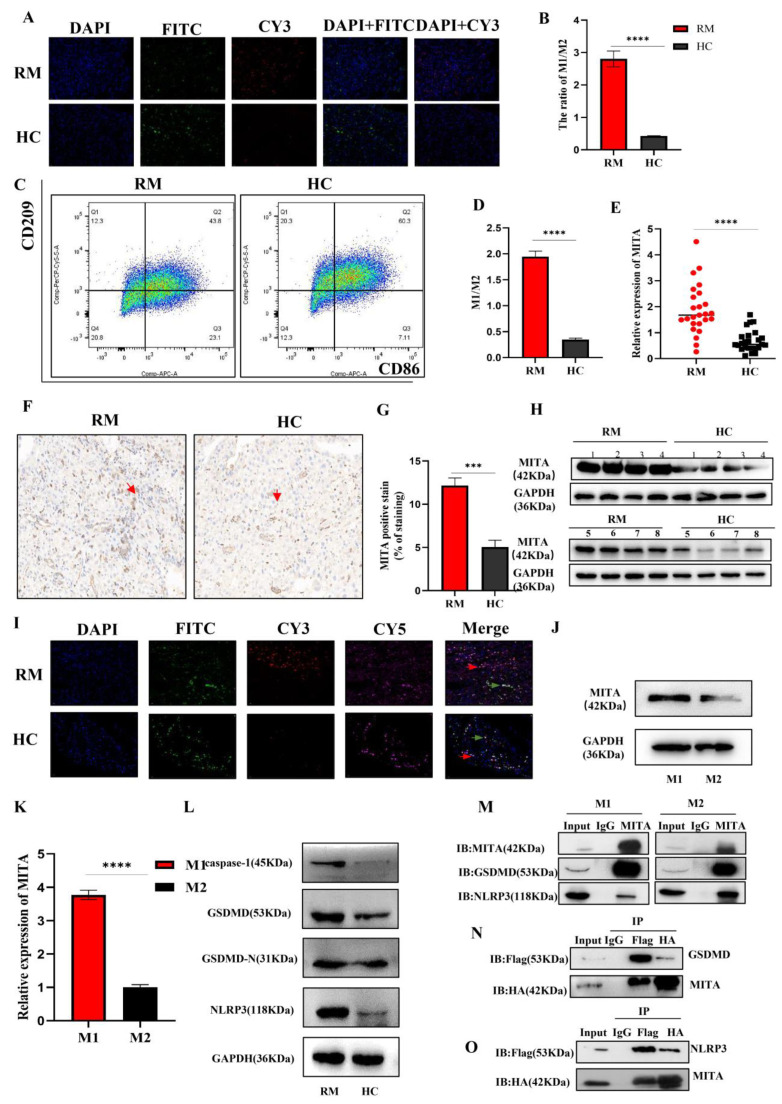
MITA is highly upregulated in decidual macrophages derived from RM patients and is related to RM. (**A**) The M1/M2 ratio evaluated by mIF in decidual tissues (50×) and the relative quantitative analysis. CD86 (CY3), CD206 (FITC), and DAPI. Scale bar = 0.020 mm. (**B**) The quantitative analysis of the M1/M2 ratio of mIF results. (**C**) The M1/M2 ratio evaluated by FCM in decidual tissues (M1 (CD86 positive cells) and M2 (CD209 positive cell)). (**D**) The quantitative analysis of the M1/M2 ratio of FCM results. (**E**) The relative expression of MITA in decidual macrophages from RM and HC patients by qRT-PCR. (**F**) IHC staining of MITA in decidual tissues derived from RM and HC patients (IHC stain, ×40). Scale bar = 0.020 mm. The red row represents the MITA positive region. (**G**) Relative quantitative analysis of the IHC results for the decidual tissues of RM and HC patients. (**H**) The relative expression of MITA in decidual macrophages from RM and HC patients by WB. (**I**) Colocalizing MITA and CD86 or CD206 by mIF in decidual tissues of RM and HC patients (50×). CD86 (CY3), CD206 (FITC), MITA (CY5), and DAPI. Scale bar = 0.020 mm. The red row represents the co-localization of MITA and CD86 in cells from decidual tissues. The green row represents the co-localization of MITA and CD206 in cells from decidual tissues. (**J**) The relative expression of MITA in M1 and M2 cells derived from TDM cells by Western blot. (**K**) The relative expression of MITA in M1 and M2 cells derived from TDM cells by WB. (**L**) The relative expression of pyroptosis-related markers (caspase-1, GSDMD, GSDMD-N, and NLRP3) in decidual macrophages in RM and HC patients by WB. (**M**) The interactive relationship between MITA and GSDMD, MITA and NLRP3 was validated in M1 and M2 cells derived from TDM cells. (**N**) The interactive relationship between MITA and GSDMD by CO-IP in HEK293T cells. (**O**) The interactive relationship between MITA and NLRP3 by CO-IP in HEK293T cells. Gene expression was normalized to GAPDH. The data are shown as fold change in RNA copies relative to the control group. Quantitative data are presented as the mean ± standard deviation of the three independent experiments. Statistical comparison between two groups was made by unpaired *t*-test. *** *p <* 0.001; **** *p <* 0.0001.

M1/M2 macrophage proportions change during different stages of pregnancy to protect the fetus. For fetomaternal tolerance to be sustained, more macrophages are polarized toward M2 macrophages. It has also been observed that abnormal pregnancies produce more M1 macrophages at the maternal–fetal interface. For the contraction of spiral arteries, invasion of trophoblasts, and phagocytosis of apoptotic cells, it is vital to maintain a balance between M1 and M2 macrophage polarizations during pregnancy [15,16]. As shown in Figure 1A,B, the mIF results demonstrated that the M1/M2 ratio in decidual tissues is higher in RM patients as compared to HC patients (The negative control staining of mIF were shown in Figure S3). Moreover, similar results were validated by FCM assay (Figure 1C,D). These results showed an imbalance of polarization between M1 and M2 macrophages in decidual tissues in RM patients, which is similar to previous findings. We then evaluated the expression of MITA in decidual macrophages derived from RM and HC patients by qRT-PCR and WB and found that MITA in the decidual macrophages was significantly upregulated in RM patients compared to HC patients (Figure 1E,H). The IHC results indicated similar results (Figure 1F,G) (The negative control staining of IHC were shown in Figure S2). Furthermore, mIF showed the colocalization of MITA and macrophage surface biomarkers, such as CD86 and CD206, in both the RM and HC decidual tissues (Figure 1I). THP-1 cells can moderately polarize to M1/M2 cells under different conditions. After cell polarization was induced, a qRT-PCR assay was conducted to detect M1 (iNOS and TNF-α) and M2 (ARG-1 and IL-10) markers. As compared to M0, the mRNA level of M1 markers (iNOS and TNF-α) was significantly upregulated in M1 cells, and the mRNA level of M2 markers (ARG-1 and IL-10) was significantly upregulated in M2 cells (Supplementary Figure S1A,B). We also evaluated MITA expression in M1 and M2 macrophages and found that MITA was significantly upregulated in M1 cells compared to M2 cells, both in protein and mRNA levels (Figure 1J,K). The removal of functionally infected or potentially neoplastic cells is driven by programmed cell death (PCD) pathways, highlighting their essential roles in host defense against pathogens, cancer, and other pathologies [17]. Among them, pyroptosis is one of the lytic forms of cell death resulting from the formation of pores or other breaches in the plasma membrane [18,19]. Mounting evidence has demonstrated that macrophages participate in many human diseases, such as sepsis-related acute lung injury [20] and periodontal inflammaging [21]. However, the functions between pyroptosis and macrophages in RM remain unclear. Next, we evaluated the pyroptosis-related markers of WB. We demonstrated that the expression of caspase-1, GSDMD, GSDMD-N, and NLRP3 was significantly upregulated in decidual macrophages in RM patients compared to HC patients (Figure 1L). To verify whether MITA could participate in pyroptosis in macrophages, endogenous IP results in M1 and M2 cells derived from TDM cells showed that MITA could interact with GSDMD and NLRP3 (Figure 1M). Further CO-IP results in HEK 293T cells also demonstrated similar results (Figure 1N,O). The above-mentioned results indicated that MITA might be a promising molecule that may participate in the pathology of RM.

### 2.2. MITA Could Promote Macrophage Apoptosis and Proinflammatory Polarization in TDM Cells

**Figure 2:**
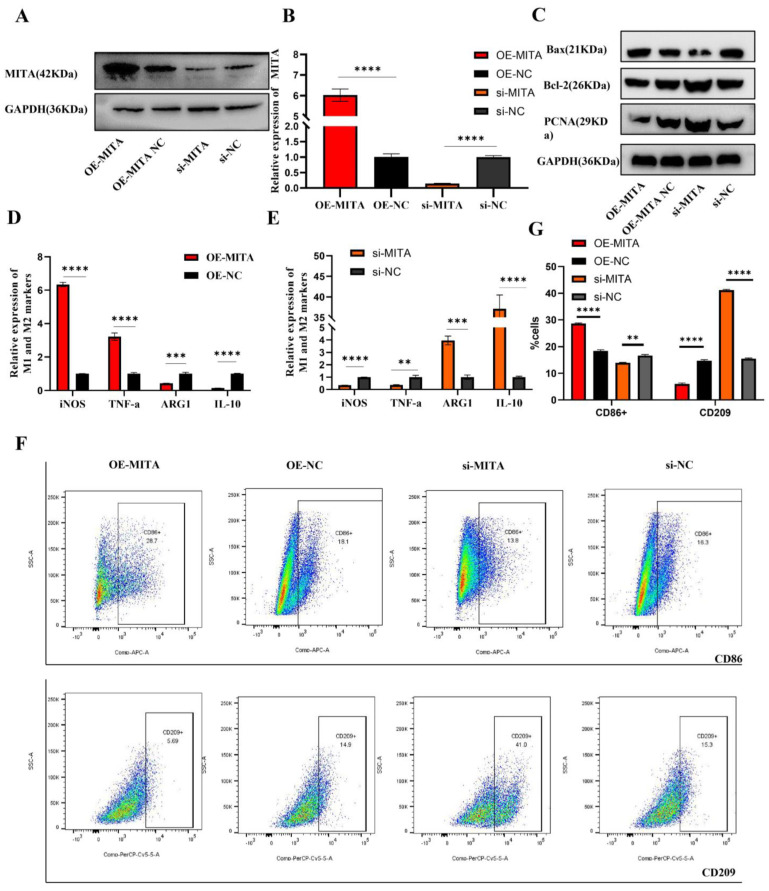
MITA promotes macrophage apoptosis and proinflammatory polarization in TDM cells. (**A**) Measurement of transfection efficiency of MITA overexpression plasmid or siRNAs was performed by Western blot. (**B**) Measurement of transfection efficiency of MITA overexpression plasmid or siRNAs was performed by qRT-PCR. (**C**) The protein levels of Bax, Bcl-2, and PCNA were detected by WB. (**D**) The mRNA marker levels of M1 and M2 were measured by qRT-PCR in TDM cells after transfecting with the MITA overexpression plasmid and its negative control. (**E**) The mRNA marker levels of M1 and M2 were measured by qRT-PCR in TDM cells after transfecting with the siRNA targeting MITA and its negative control. (**F**) The proportions of M1 (CD86 positive) and M2 (CD209 positive) cells were measured by FCM. (**G**) The quantitative results of the FCM results in TDM cells after transfecting with MITA overexpression plasmid or siRNAs and their negative controls. Gene expression was normalized to GAPDH. The data are shown as fold change in RNA copies relative to the control group. Quantitative data are presented as the mean ± standard deviation of the three independent experiments. Statistical comparison between two groups was made by unpaired *t*-test. ** *p <* 0.01; *** *p <* 0.001; **** *p <* 0.0001.

To test our hypothesis that MITA is involved in macrophage polarization and programmed cell death, we used MITA expression plasmid or siRNAs to transiently overexpress or silence gene expression and subjected cells to qPCR, Western blot and flow cytometry assays. Then, the overexpression or reduction of MITA was validated in mRNA, and the protein levels in TDM cells were assessed (Figure 2A,B). Next, we detected the expression of apoptosis-related protein and proliferation-related markers. The results manifested that the overexpression of MITA promoted cell apoptosis while repressing cell proliferation in TDM cells (Figure 2C), and silencing the expression of MITA had the opposite effects (Figure 2C). Then, we further examined the macrophage polarization markers by qRT-PCR and FCM assays. We found that the overexpression of MITA dramatically promoted the M1 markers (iNOS and TNF-α) while reducing the M2 markers (ARG-1 and IL-10) (Figure 2D). Furthermore, silencing the expression of MITA had the opposite effect on the expression of M1 and M2 markers (Figure 2E). Furthermore, we found that the overexpression of MITA increased the proportion of CD86-positive cells while reducing the proportion of CD209-positive cells in TDM cells (Figure 2G), However, silencing the expression of MITA increased the proportion of CD209-positive cells while reducing the proportion of CD86-positive cells in TDM cells (Figure 2G). The quantitative analysis of CD86-positive or CD209-positive cells according to flow cytometry results is shown in Figure 2F. These results indicated that MITA markedly promoted macrophage apoptosis and proinflammatory polarization in TDM cells.

### 2.3. Expression Pattern of circRNAs in Decidual Macrophages

**Figure 3:**
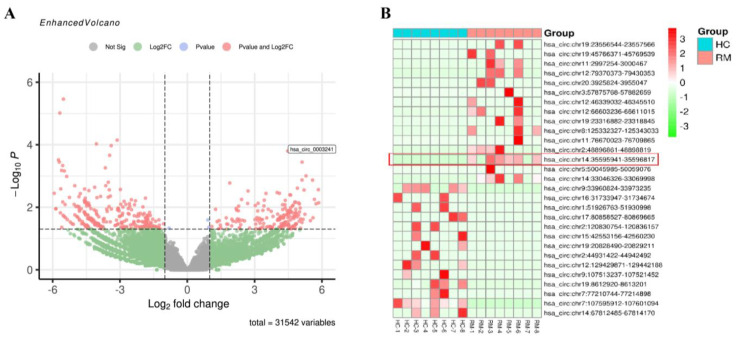

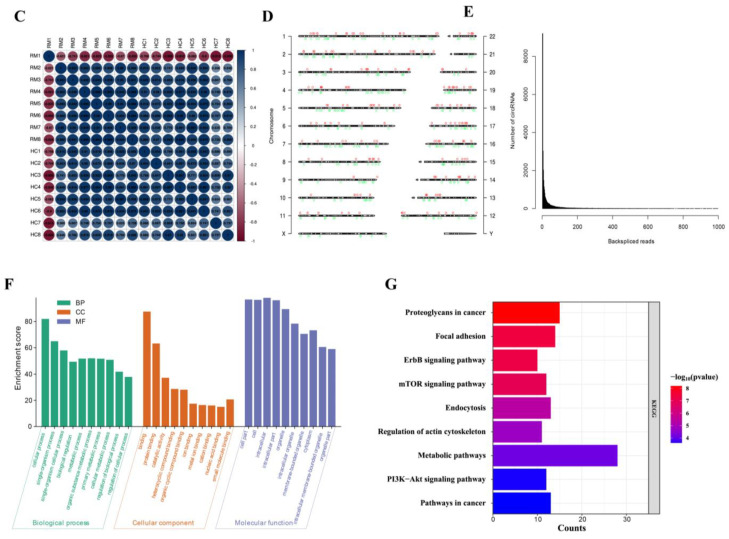
The circRNA expression profiles in decidual macrophages and bioinformatic analysis. (**A**) Volcano map showing the differences in the expression of circRNAs in decidual macrophages derived from the RM and HC subjects. (**B**) Hierarchical clustering of the differentially expressed circRNAs. The red dots represent the upregulated circRNAs, and the green dots represent the downregulated circRNAs. The molecule represented within the red box is the target of this study. (**C**) Spearman’s rank correlation tests evaluated the correlation rank between RM and HC patients. (**D**) Chromosome distribution of differentially expressed circRNAs. The red dots represent the upregulated circRNAs, and the green dots represent the downregulated circRNAs. (**E**) The number of circRNAs and back-spliced reads identified in RM and HC patients. (**F**) GO enrichment of the parental mRNAs of the differentially expressed circRNAs. (**G**) KEGG enrichment of the parental mRNAs of the differentially expressed circRNAs.

So, what causes the high expression of MITA in RM? To elucidate this, the expression profiles of decidual macrophages derived from eight RM and eight corresponding HC women were detected using circRNA high-throughput sequencing analysis. The total RNAs derived from decidual macrophages were subjected to linear RNA removal via ribosomal RNA (rRNA) depletion and RNaseR treatment to enrich circRNAs. A total of 479 circRNA targets, including 196 upregulated and 283 downregulated circRNAs, were detected in eight pairs of subjects. A volcano plot was generated to visualize the differential expression between RM and HC patients (Figure 3A). The top 15 upregulated and downregulated circRNAs were shown by hierarchical clustering (Figure 3B), and their basal characteristics are presented in Table 1. Figure 3C shows a correlation heatmap by Spearman’s rank correlation between samples. Figure 3D shows the distribution map of differentially expressed circRNAs in different chromosomes. Figure 3E shows the number of circRNAs and back-spliced reads identified in the RM and HC patients (Figure 3E). To speculate on the potential circRNA functions, GO and KEGG pathway enrichment analyses of all of the differentially expressed circRNA host genes were performed. For BP, the significantly enriched GO terms for all of the differentially expressed circRNA host genes were cellular process, single-organism process, single-organism cellular process, organic substance metabolic process, and metabolic process. For CC, intracellular, cell part, cell, and organelle were significantly enriched. Meanwhile, binding, protein binding, catalytic activity, heterocyclic compound binding, and organic cyclic compound binding were enriched for MF (Figure 3F). KEGG pathway analysis revealed that the host genes of these differentially expressed circRNAs might be involved in proteoglycans in cancer, focal adhesion, the ErbB signaling pathway, Fc GAMMA R-mediated phagocytosis, and the cell cycle (Figure 3G).

### 2.4. Decidual Macrophage circKIAA0391 Is Upregulated and May Participate in RM

**Figure 4:**
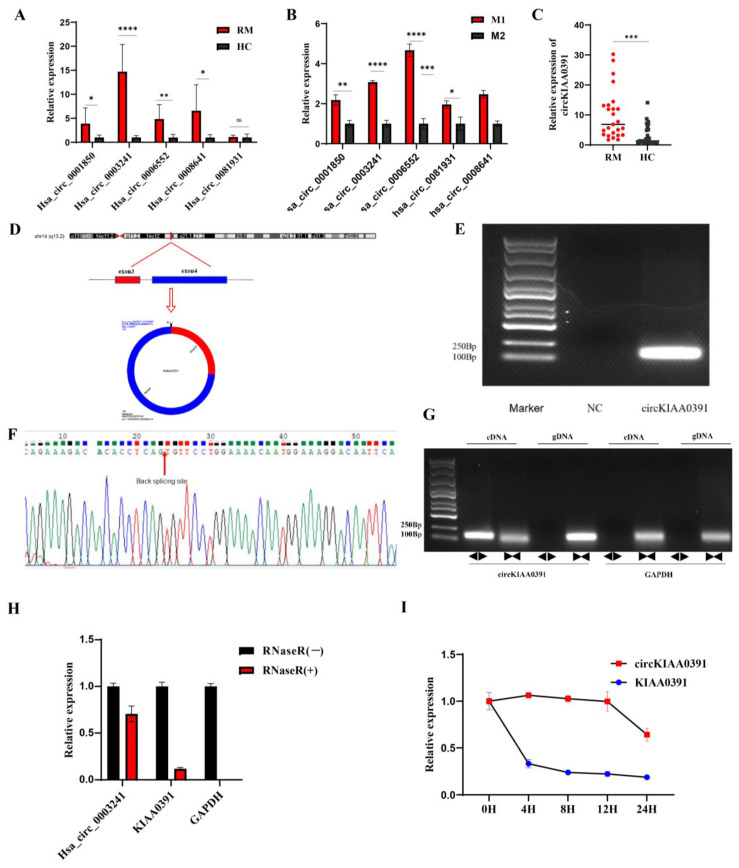
Decidual macrophage circKIAA0391 was upregulated and may participate in RM. (**A**) The circRNA expression was validated by qRT-PCR in decidual macrophages derived from RM and HC patients (n = 8 for each group). (**B**) The circRNA expression was validated by qRT-PCR in M1 and M2 cells derived from TDM cells. (**C**) Expression of circKIAA0391 in decidual macrophages derived from RM and HC patients (n = 25 for each group). (**D**) The spliced mature sequence length of circKIAA0391 originated from the KIAA0391 gene. (**E**) PCR amplification and agarose gel electrophoresis assay. (**F**) Sanger sequence verification using divergent primers. (**G**) The PCR products amplified using divergent or convergent primers were verified by agarose gel electrophoresis. A represents divergent primers, and B represents convergent primers. (**H**) qRT-PCR was used to analyze the expression of circKIAA0391, KIAA0391, and GAPDH after the treatment with RNaseR. (**I**) The expression of circKIAA0391 and KIAA0391 was measured by qRT-PCR in TDM cells after Act-D and DMSO treatment. Gene expression was normalized to GAPDH. The data are shown as fold change in RNA copies relative to the control group. Quantitative data are presented as the mean ± standard deviation of the three independent experiments. Statistical comparison between two groups was made by unpaired *t*-test. * *p <* 0.05; ** *p <* 0.01; *** *p <* 0.001; **** *p <* 0.0001; ns indicates no significance.

Next, we selected the top five circRNAs (Hsa_circ_0001850, Hsa_circ_0003241, Hsa_circ_0006552, Hsa_circ_0008641, and Hsa_circ_0081931) from the upregulated and downregulated circRNAs for further evaluation. The selected circRNAs were validated in decidual macrophages from these eight pairs of microarray cohorts by qRT-PCR, and we found that except for Hsa_circ_0081931, the other four circRNAs showed similar dysregulated expression as compared to the microarray results (Figure 4A). Furthermore, we found that all of the circRNAs were significantly upregulated in M1 cells compared to M2 cells (Figure 4B). The results for Hsa_circ_0003241, which had higher expression in the RM and M1 cells compared to the HC and M2 cells, were mainly consistent with the microarray data. We then enlarged the sample size for qRT-PCR validation and found that Hsa_circ_0003241 was stably upregulated in 25 RM patients as compared to the HC patients (Figure 4C). Thus, we speculated that Hsa_circ_0003241 in the decidual macrophages might be involved in the pathogenesis of RM. 

According to the circBase database and UCSC genome browser (http://genome.ucsc.edu/ (accessed on 20 September 2021)), Hsa_circ_0003241 is derived from exons 3 and 4 of KIAA0391, which is located on chromosome 14 and has a length of 181 nucleotides. We then changed the name of Hsa_circ_0003241 to circKIAA0391 (circBank ID: hsa_circKIAA0391_001). The ring formation mechanism and the constitution characteristics of the host gene (KIAA0391) are shown in Figure 4D. We amplified circKIAA0391 by PCR with the designed divergent primers. The agarose gel electrophoresis results showed that circKIAA0391 had a single band at the expected location (Figure 4E). Then, the back-splicing junction was validated by Sanger sequencing of the PCR product, which was consistent with that in the circBase database (Figure 4F). To verify the circular characteristics of circKIAA0391, we designed divergent primers to amplify the circKIAA0391 form and convergent primers to amplify another exon of the linear KIAA0391 mRNA. The products amplified using divergent or convergent primers were then verified by agarose gel electrophoresis. We found that divergent primers could amplify circKIAA0391 in cDNA but not gDNA and that it could be amplified by convergent primers in cDNA and gDNA, whereas GAPDH could be amplified only using convergent primers in both cDNA and gDNA (Figure 4G). These results suggested that circKIAA0391 is a circRNA generated from back-splicing of the KIAA0391 gene transcript but not genetic recombination. Anti-RNaseR digestion tests showed that circKIAA0391 was resistant to RNaseR, while liner KIAA0391 and GAPDH were not (Figure 4H). The half-life of circKIAA0391 in Act-D treated TDM cells exceeded 24 h, whereas the corresponding linear KIAA0391 had a half-life of less than 4 h (Figure 4I). These results confirmed the stable circular structure of circKIAA0391.

### 2.5. circKIAA0391 Promotes TDM Cell Apoptosis and Proinflammatory Polarization

**Figure 5:**
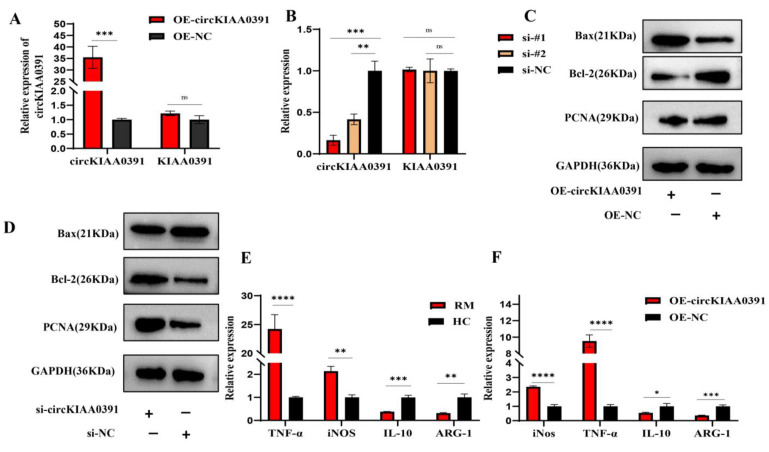

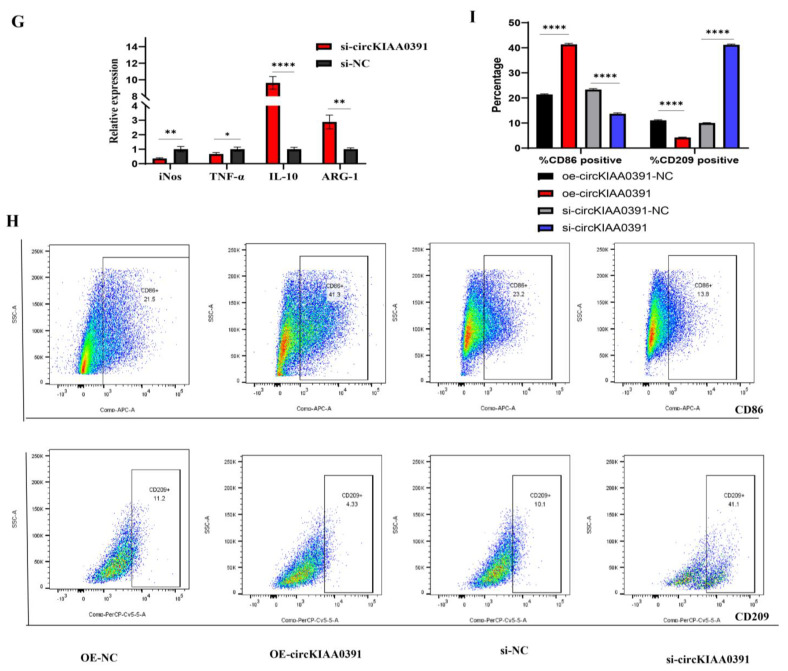
circKIAA0391 promoted TDM cell apoptosis and proinflammatory polarization. (**A**) The expression of circKIAA0391 and KIAA0391 in TDM cells after lentivirus infection was measured by qRT-PCR. (**B**)The expression of circKIAA0391 and KIAA0391 was measured in TDM cells after siRNA transfection by qRT-PCR. (**C**,**D**) circKIAA0391 influenced the protein expression of the Bax, Bcl-2, and PCNA as detected by WB. (**E**) The mRNA marker levels of M1 and M2 were measured by qRT-PCR in decidual macrophages derived from RM and HC patients. (**F**) The mRNA marker levels of M1 and M2 were measured by qRT-PCR in TDM cells after lentivirus infection for circKIAA0391 overexpression and negative control. (**G**) The mRNA marker levels of M1 and M2 were measured by qRT-PCR in TDM cells after siRNA transfection for silencing the expression of circKIAA0391 and its negative control. (**H**) The proportion of M1 (CD86 positive) and M2 (CD209 positive) cells was measured by FCM in TDM cells after lentivirus infection or siRNA transfection and their negative control. (**I**) The quantitative results of the FCM results in TDM cells after lentivirus infection or siRNA transfection and their negative control. Gene expression was normalized to GAPDH. The data are shown as fold change in RNA copies relative to the control group. Quantitative data are presented as the mean ± standard deviation of the three independent experiments. Statistical comparison between two groups was made by unpaired *t*-test. * *p <* 0.05; ** *p <* 0.01; *** *p <* 0.001; **** *p <* 0.0001; ns indicates no significance.

To better understand the functions of circKIAA0391, lentivirus containing the OE-circKIAA0391, or the siRNAs targeting the back-splicing site, were designed and transfected into TDM cells. The qRT-PCR results showed that the expression of circKIAA0391 was significantly increased in the OE-circKIAA0391 group compared to the control group. However, there was no significant difference in the expression of its linear parental mRNA KIAA0391 (Figure 5A). Since only the back splice junction was a unique sequence to distinguish the circRNA from its linear counterpart, we designed two siRNA sequences targeting the back splice site. The results demonstrated that the two siRNAs could reduce the expression of circKIAA0391 but not KIAA0391 (Figure 5B). The silencing efficiency of si-#1 was much better than that of si-#2, and we thus selected si-#1 for further research. To better understand the effect of circKIAA0391 on apoptosis, we evaluated the expression of apoptotic proteins. We found that the overexpression of circKIAA0391 inhibited the viability and promoted the apoptosis of TDM cells, while silencing circKIAA0391 had the opposite effects (Figure 5C,D). Due to the critical roles of macrophage polarization balance in RM, we further evaluated whether circKIAA0391 could influence macrophage polarization in vivo. Furthermore, we found that M1 macrophage polarization marker genes (TNF-α and iNOS) were significantly upregulated, while M2 macrophage polarization marker genes (IL-10 and ARG-1) were significantly downregulated from the decidual macrophages in the RM patients as compared to the HC patients (Figure 5E). Then, the mRNA expression of M1 markers was upregulated or downregulated after the overexpression or silencing of circKIAA0391; however, the opposite results were found in M2 markers (Figure 5F,G). 

Then, the extent of macrophage phenotypic polarization was investigated using flow cytometry. We found that the CD86-positive cells (M1 cells) were significantly upregulated or downregulated, respectively, after we overexpressed or silenced the expression of circKIAA0391 in TDM cells; however, the opposite results were shown for the CD209-positive cells (M2 cells) (Figure 5H,I). These findings indicated that circKIAA0391 could promote cell apoptosis and proinflammatory polarization of TDM cells.

### 2.6. CircKIAA0391 Acts as a Sponge for miR-512-5p

**Figure 6:**
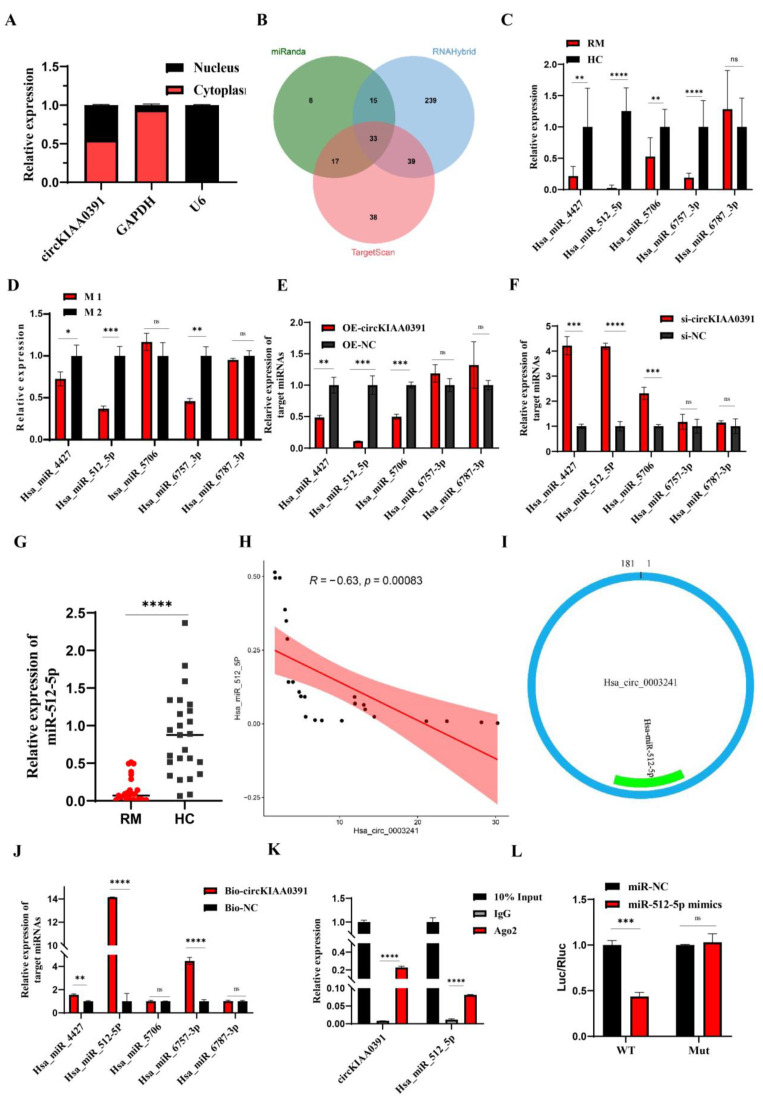
CircKIAA0391 as a sponge for miR-512-5p. (**A**) The circKIAA0391 expression in the cytoplasm and nucleus of TDM cells was analyzed by qRT-PCR. (**B**) Venn diagram of overlapped miRNA results predicted by miRanda, RNAHybrid, and TargetScan. (**C**) The relative expression of five candidate miRNAs was validated in the microarray cohorts by qRT-PCR (n = 8). (**D**) The relative expression of five candidate miRNAs was validated in the M1 and M2 cells derived from TDM cells by qRT-PCR. (**E**) The expression of five candidate miRNAs was validated by qRT-PCR in TDM cells after lentivirus infection for circKIAA0391 overexpression and negative control. (**F**) The expression of five candidate miRNAs was validated by qRT-PCR in TDM cells after siRNA transfection and its negative control. (**G**) The relative expression of miR-512-5p was validated in the validation cohorts by qRT-PCR (n = 25, respectively). (**H**) Pearson correlation analysis between miR-512-5p and circKIAA0391 in decidual macrophages of RM patients. (**I**) The prediction results for the binding site with miR-512-5p on circKIAA0391 by the miRanda software. (**J**) The relative expression levels of the five candidate miRNAs were detected by biotin-labeled circRNA pull-down assay. (**K**) The relative expression of circKIAA0391 and miR-512-5p was detected by the anti-AGO2 RIP assay. (**L**) Luciferase reporter assay for the luciferase activity of wild-type and mutant circKIAA0391 vectors co-transfected with miR-512-5p mimics or mimics of NC. Gene expression was normalized to GAPDH. The data are shown as fold change in RNA copies relative to the control group. Quantitative data are presented as the mean ± standard deviation of the three independent experiments. Statistical comparison between two groups was made by unpaired *t*-test. * *p <* 0.05; ** *p <* 0.01; *** *p <* 0.001; **** *p <* 0.0001; ns indicates no significance.

It is well known that the subcellular localization of circRNA determines its function. Thus, we performed the nuclear and cytoplasmic separation assay to identify the subcellular location of circKIAA0391 in TDM cells. As shown in Figure 6A, the nuclear and cytoplasmic separation assay demonstrated that circKIAA0391 predominantly localized in the cytoplasm. Since the circRNA-mediated ceRNA mechanism requires circRNA and miRNA to co-exist in the cytoplasm, we speculated that circKIAA0391 might act as an miRNA sponge to regulate gene expression. Then, three independent miRNA target databases were used to explore the binding miRNAs of circKIAA0391. As shown in Figure 6B, after taking the intersection of the prediction results from three databases, there were 33 candidate miRNAs. The prediction results are shown in Table 2 (miRanda software v3.3a). According to the results above and related references, we randomly chose five miRNAs (miR-4427, miR-512-5p, miR-5706, miR-6757-3p, and miR-6787-3p) for further research. Firstly, the selected miRNAs were validated in the microarray cohorts (Figure 6C), and we found that except for miR-6787-3p, the expression of the other four candidate miRNAs was significantly downregulated in the decidual macrophages of RM patients. We utilized the M1 and M2 cells to verify their expression (Figure 6D), and the results were consistent with the qRT-PCR results from the microarray cohorts. As shown in Figure 6E,F, the overexpression of circKIAA0391 in TDM cells reduced the expression of miR-4427, miR-512-5p, and miR-5706, while silencing the expression of circKIAA0391 had the opposite effects in TDM cells. Therefore, we found that miR-512-5p had the most potential binding ability to circKIAA0391. Then, we further examined the expression level of miR-512-5p in the whole cohort and found that miR-512-5p was significantly reduced in decidual macrophages from RM patients compared to the HC patients (Figure 6G). Pearson correlation analysis demonstrated that the expression of miR-512-5p was inversely correlated with circKIAA0391 in TDM cells (Figure 6H). The binding site predicted by miRanda software is visualized in Figure 6I. After pulling down by biotin-labeled circKIAA0391 and oligo probes, the five candidate miRNAs were investigated by qRT-PCR. The outcomes revealed that the expression of miR-512-5p was significantly enriched by the biotin-labeled circKIAA0391 probe (Figure 6J). The RIP results showed that circKIAA0391 and miR-512-5p pulled down by anti-AGO2 antibodies were enriched considerably, suggesting that circKIAA0391 and miR-512-5p existed in RISCs (Figure 6K). We performed a dual luciferase reporter assay to verify the binding site of circKIAA0391 and miR-512-5p. The results revealed that the relative luciferase activity was significantly decreased in HEK 293T cells co-transfected with miR-512-5p mimics and reporter constructs carrying wild-type binding sequences. In contrast, no luciferase activity changes were found in cells co-transfected with miR-512-5p mimics and reporter constructs carrying mutant binding sites of 3′-UTR of circKIAA0391 (Figure 6L). Taking these findings together, we identified that circKIAA0391 acts as a sponge for miR-512-5p.

### 2.7. MITA Is the Target of miR-512-5p

**Figure 7:**
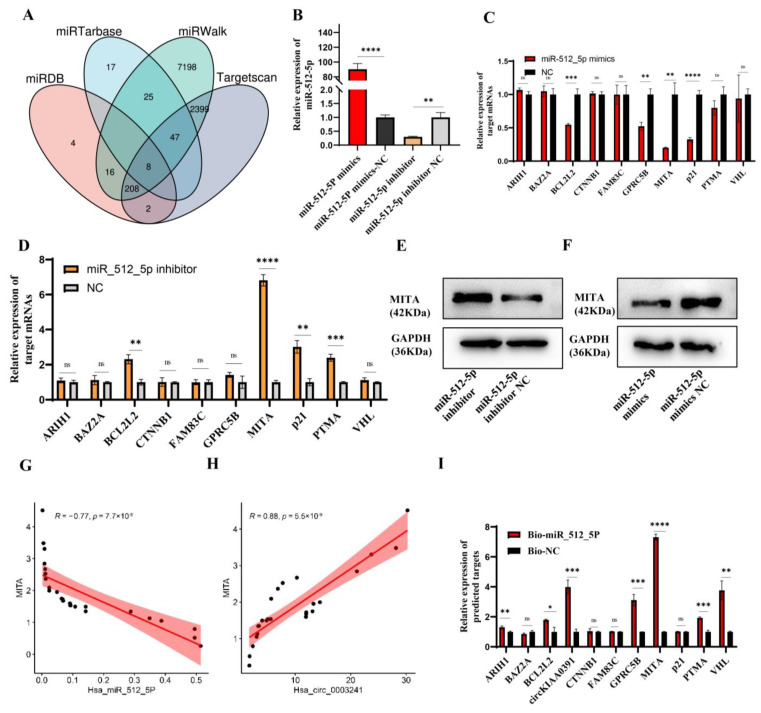
MITA is the target of miR_512_5p. (**A**) Venn diagram showing overlapped mRNAs predicted from miRDB, miRTarbase, miRWalk, and TargetScan databases. (**B**) miR-512-5p mimics or miR-512-5p inhibitors were transfected in TDM cells, and their overexpression or silencing efficiency was verified by qRT-PCR. (**C**) The expression of the 10 potential mRNA targets in TDM cells after transfecting with miR-512-5p mimics and its negative control was detected by qRT-PCR. (**D**) The expression of the 10 potential mRNA targets in TDM cells after transfecting miR-512-5p inhibitor and its negative control was detected by qRT-PCR. (**E**) The protein level of MITA was measured by WB in TDM cells after transfecting with miR-512-5p mimics and its negative control. (**F**) The protein level of MITA was measured by WB in TDM cells after transfecting with miR-512-5p inhibitor and its negative control. (**G**) Pearson correlation analysis between MITA and miR-512-5p in decidual macrophages in RM patients. (**H**) Pearson correlation analysis between MITA and circKIAA0391 in decidual macrophages in RM patients. (**I**) The expression of the 10 potential mRNA targets was detected by biotin-labeled miRNA pull-down assay. Gene expression was normalized to GAPDH. The data are shown as fold change in RNA copies relative to the control group. Quantitative data are presented as the mean ± standard deviation of the three independent experiments. Statistical comparison between two groups was made by unpaired *t*-test. * *p <* 0.05; ** *p <* 0.01; *** *p <* 0.001; **** *p <* 0.0001; ns indicates no significance.

To further investigate the functions of miR-512-5p, we utilized midrib, miRTarbase, miRWalk, and Targetscan databases to predict the potential binding mRNAs and found eight overlapped mRNAs (ARIH1, BCL2L2, GPRC5B, CTNNB1, FAM83C, PTMA, BAZ2A, and VHL) (Figure 7A). By searching the website, we further included p21 as the potential target of miR-512-5p in TDM cells, as this binding relationship had been validated in non-small cell lung cancer [22,23]. Due to the close relationship between MITA and RM in decidual macrophages, we further evaluated whether the circKIAA0391–miR-512-5p axis could impact the expression of MITA. In TDM cells, we transfected miR-512-5p mimics or inhibitors to verify their regulation of miRNAs.

Firstly, we confirmed the transfection efficiency using qRT-PCR (Figure 7B). As shown in Figure 7C,D, the qRT-PCR results showed that MITA was the most significantly downregulated when transfected with miR-512-5p mimics, while it was upregulated when transfected with miR-512-5p inhibitors. Furthermore, similar results were found in MITA protein levels (Figure 7E,F). Pearson correlation analysis revealed the negative correlation between MITA and miR-512-5p (R = −0.77, *p*-value = 7.7 × 10^−6^) and a positive correlation between MITA and circKIAA0391 (R = 0.88, *p*-value = 5.5 × 10^−9^) (Figure 7G,H). Furthermore, biotinylated miRNA pull-down assays were performed after transfecting biotinylated miR-512-5p mimic or biotinylated non-targeting miRNA into the TDM cells. We found that MITA was significantly enriched (Figure 7I). These results showed that MITA was the target of miR-512-5p.

### 2.8. CircKIAA0391 Regulates TDM Cell Apoptosis and Polarization by Targeting the miR-512-5p/MITA Axis

**Figure 8:**
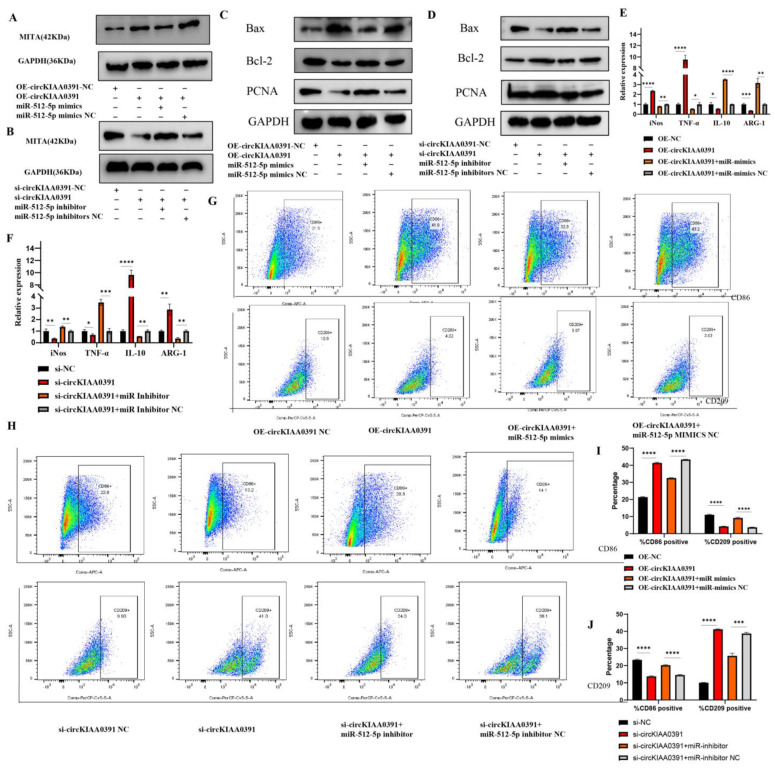
CircKIAA0391 regulates apoptosis and proinflammatory polarization in TDM cells by targeting the miR-512-5p/MITA axis. (**A**) The protein levels of MITA were detected in TDM cells after transfecting with oe-circKIAA0391 NC, oe-circKIAA0391, oe-circKIAA0391 + miR-512-5p mimics, and oe-circKIAA0391 + miR-512-5p mimic NC. (**B**) The protein levels of MITA were detected in TDM cells after transfecting with si-circKIAA0391 NC, si-circKIAA0391, si-circKIAA0391 + miR-512-5p inhibitor, and si-circKIAA0391 + miR-512-5p inhibitor NC. (**C**) The protein levels of Bax, Bcl-2, and PCNA were detected in TDM cells after transfecting with oe-circKIAA0391 NC, oe-circKIAA0391, oe-circKIAA0391 + miR-512-5p mimics, and oe-circKIAA0391+ miR-512-5p mimic NC. (**D**) The protein levels of Bax, Bcl-2, and PCNA were detected in TDM cells after transfecting with si-circKIAA0391 NC, si-circKIAA0391, si-circKIAA0391 + miR-512-5p inhibitor, and si-circKIAA0391 + miR-512-5p inhibitor NC. (**E**) The mRNA markers of M1 and M2 were detected by qRT-PCR in TDM cells after transfecting with oe-circKIAA0391 NC, oe-circKIAA0391, oe-circKIAA0391 + miR-512-5p mimics, and oe-circKIAA0391 + miR-512-5p mimic NC. (**F**) The markers of M1 and M2 were detected by qRT-PCR in TDM cells after transfecting with si-circKIAA0391 NC, si-circKIAA0391, si-circKIAA0391 + miR-512-5p inhibitors, and si-circKIAA0391 + miR-512-5p inhibitor NC. (**G**) The proportions of M1 (CD86 positive) and M2 (CD209 positive) cells were measured by FCM in TDM cells after transfecting with oe-circKIAA0391 NC, oe-circKIAA0391, oe-circKIAA0391 + miR-512-5p mimics, and oe-circKIAA0391 + miR-512-5p mimic NC. (**H**) The proportions of M1 (CD86 positive) and M2 (CD209 positive) cells were measured by FCM in TDM cells after transfecting with si-circKIAA0391 NC, si-circKIAA0391, si-circKIAA0391 + miR-512-5p inhibitor, and si-circKIAA0391 + miR-512-5p inhibitor NC. (**I**) The quantitative results of the FCM results in Figure 6G. (**J**) The quantitative results of the FCM results in Figure 6H. Gene expression was normalized to GAPDH. The data were shown as fold change in RNA copies relative to the control group. Quantitative data are presented as the mean ± standard deviation of the three independent experiments. Statistical comparison between two groups was made by unpaired *t*-test. * *p* < 0.05; ** *p* < 0.01; *** *p* < 0.001; **** *p* < 0.0001; ns indicates no significance.

To confirm whether circKIAA0391 regulates TDM cell apoptosis and proinflammatory polarization by sponge activity of miR-512-5p, consequently upregulating MITA, miR-512-5p mimics and/or inhibitors were used to execute rescue experiments. As shown in Figure 8A, miR-512-5p mimics could significantly reduce the upregulation of MITA protein expression induced by overexpression of circKIAA0391. More importantly, silencing circKIAA0391 could reduce the expression of MITA protein levels, which was reversed by miR-512-5p mimics (Figure 8B). These data convincingly demonstrated that MITA is a direct target of circKIAA0391/miR-512-5p. We call it the circKIAA0391/miR-512-5p/MITA axis. Then, we validated whether the circKIAA0391/miR-512-5p axis could regulate TDM cell apoptosis and proinflammatory polarization by sponging MITA. As shown in Figure 8C, miR-512-5p mimics significantly blocked the circKIAA0391 overexpression-mediated promotion of apoptosis and inhibition of proliferation in TDM cells. Meanwhile, miR-512-5p inhibitors significantly blocked the circKIAA0391 silencing, mediating the promotion of proliferation and inhibition of apoptosis (Figure 8D). As shown in Figure 8E, miR-512-5p mimics significantly blocked the circKIAA0391 overexpression-mediated increase in M1 markers and decrease in M2 markers. On the other hand, the miR-512-5p inhibitor could dramatically block the circKIAA0391 silencing-induced increase in M2 markers and decrease in M1 markers (Figure 8F). Furthermore, the FCM results showed that miR-512-5p mimics could significantly block the circKIAA0391 overexpression, inducing the promotion of CD86-positive cells and the reduction of CD209-positive cells (Figure 8G,I). Meanwhile, promoting CD209-positive cells and reducing CD86-positive cells induced by circKIAA0391 silencing could be blocked by miR-512-5p (Figure 8H,J). Therefore, we confirmed that the circKIAA0391/miR-512-5p axis could regulate TDM cell apoptosis and macrophage proinflammatory polarization by targeting MITA.

## 3. Discussion

RM is a common pregnancy complication with a multi-factorial etiology, representing a global public health challenge. As noted, MITA has been associated with infection as well as autoinflammatory, autoimmune, and degenerative diseases [7,24]. However, little is known about the expression of MITA in macrophages in RM. In this study, we first found that the ratio of M1/M2 in decidual tissues of RM patients was significantly higher than that in HC patients. We then found that the expression of MITA was upregulated in RM patients and M1 cells derived from TDM cells, as compared to the HC patients and M2 cells derived from TDM cells, respectively. Further results also demonstrated that MITA could directly interact with GSDMD or NLRP3 in TDM cells. These aforementioned results suggested that the high expression of MITA in decidual macrophages was closely related to the pathology of RM.

Recently, a body of evidence has demonstrated that MITA could act as an miRNA sponge to regulate the progression of many diseases. Chu, Q. et al. found that circRasGEF1B can bind to miR-21-3p directly and regulates MITA expression to enhance antiviral immunity in lower vertebrates [25]. Yarbrough, M.L., et al. found that miR-576-3p is induced by IRF3 concomitantly with interferon and targets STING, MAVS, and TRAF3, which are critical factors for interferon [26]. Furthermore, miR-181a could downregulate STING expression to suppress the activation of intrinsic interferons and may serve as a promising target for therapeutic exploitation in DNA oncoviruses [27]. We hypothesized that the functions of MITA in macrophages might be regulated by non-coding RNAs (ncRNAs) in RM. In the present study, we first constructed differentially expressed circRNA profiles in the decidual macrophages of RM patients compared to the HC subjects. Then, we validated that circKIAA0391 was significantly upregulated in RM patients and M1 macrophages. In vivo studies also demonstrated that circKIAA0391 promotes macrophage apoptosis and proinflammatory polarization, indicating that circKIAA0391 in decidual macrophages might participate in the pathology of RM. 

To our knowledge, exonic circRNAs are mainly located in the cytoplasm and could regulate gene expression in mammals at the transcriptional or posttranscriptional level by interacting with miRNAs [28]. CircVAPA could activate the phosphoinositide 3-kinase (PI3K)/protein kinase B (AKT) signaling pathway by modulating the miR-377-3p and miR-494-3p/insulin-like growth factor 1 receptor (IGF1R) axis to accelerate small cell lung cancer progression [29]. Circ_0088194 could promote rheumatoid arthritis fibroblast-like synoviocytes (RA-FLSs) invasion and migration via the miR-766-3P/Matrix Metalloproteinase 2 (MMP2) axis [30]. Furthermore, few studies have focused on the relationship between circRNA and miscarriage. Zhu et al. demonstrated that circPUM1 could impair the occurrence and development of RM by facilitating trophoblast cellular processes and protecting against inflammation via the miR-30a-5p/JUNB axis [31]. Su et al. found that circCYP21A1 could play a role in RM by impairing the balance of cell proliferation and apoptosis by sponging miR-224, thereby regulating PRLR [32]. However, the specific mechanism of circKIAA0391 in TDMs for RM still needs to be elucidated. In the present study, we identified that circKIAA0391 was mainly located in the cytoplasm by nucleic and cytoplasmic fractionation assay. Then, we further confirmed the direct binding relationship between miR-512-5p and circKIAA0391 by RIP, RNA pull-down, and dual-luciferase reporter assay. 

Recently, evidence has been found that circRNA could act as ceRNA in TDM cells, influencing many diseases. Hsa_circ_0005567 overexpression promoted M2 macrophage polarization by binding to the miR-492/SOCS2 axis to reduce chondrocyte apoptosis, which could inhibit the progression of osteoarthritis [33]. Zhang, C. et al. found that circPPM1F could act as a novel regulator of M1 macrophage activation through the circPPM1F-HuR-PPM1F-NF-κB axis [34]. Mounting evidence indicates that miR-512-5p can sponge many circRNAs or LncRNAs to exert its degradation effect on target mRNAs, thus leading to the different phenotypes of cells. Circ_0000215 could exert oncogenic effects in nasopharyngeal carcinoma through the miR-512-5p/PIK3R1 axis [35]. CircRPPH1 could act as an oncogene and regulate the progression of breast cancer via the circRPPH1-miR-512-5p-STAT1 axis [36]. As we know, macrophages are essential for establishing and maintaining pregnancy by blood vessel remodeling, immune tolerance, immunomodulation of maternal decidual lymphocytes, and parturition initiation [37]. Further studies have demonstrated that macrophages were skewed toward the M1 phenotype in spontaneous abortion [38]. Moreover, the overexpression of circPPM1F could promote pancreatic islet injury by enhancing M1 macrophage activation, and circPPM1F may serve as a novel potential therapeutic target for type 1 diabetes in children [34]. To our knowledge, this was the first study to investigate the binding relationship between circKIAA0391/miR-512-5p and MITA. In this study, we ascertained the regulatory network of circKIAA0391/miR-512-5p on MITA by biotin-labeled miRNA pull-down assay. Mechanistically, the results also demonstrated that circKIAA0391 could regulate the expression of MITA by binding to miR-512-5p, thus mediating cell apoptosis and proinflammatory polarization in TDM cells. This study has provided a new perspective in research on the ceRNA regulatory network for MITA in TDM cells and a scientific basis for novel developments in the diagnosis and treatment of RM.

However, there are some shortcomings in the study to be acknowledged. Firstly, according to the nuclear and cytoplasm fractionation assay, about 40% of the expression of circKIAA0391 was distributed in the nuclear fraction. However, we only investigated the functions of circKIAA0391 in the cytoplasm in the present study; whether circKIAA0391 could regulate TDM cell apoptosis and macrophage proinflammatory polarization through other mechanisms, such as interacting with RNA-binding proteins and sponging trans-acting elements, requires further investigation. Furthermore, the mechanism of circKIAA0391/miR-512-5p/MITA on macrophage was validated in cell models. Further animal experiments and in vitro experiments are required to determine the mechanism.

## 4. Materials and Methods

### 4.1. Clinical Specimens and Ethics Statements

Uterine decidual tissues were collected from 25 healthy women and 25 patients with RM at Renmin Hospital of Wuhan University (Wuhan, China) between January 2018 and December 2021. When two or more pregnancies are lost sequentially before 20 weeks of gestation, it is referred to as RM. A group called HC included women with normal, early pregnancies who faced unwanted pregnancies and ended them by artificial abortion. Participants were excluded if they met any of the following conditions: 1. symptoms of endocrine or metabolic diseases, 2. abnormal karyotype analysis, and 3. uterine abnormality confirmed by pelvic examination or ultrasound. The basal characteristics of the included patients are listed in Table 3. Decidual tissues were washed with iced phosphate buffer saline (PBS). Then, the tissues were ground to prepare the cell suspension. Monocytes were isolated from cell suspensions by density gradient centrifugation. After purification with anti-CD14-coated micro-beads (Thermofisher Scientific, Waltham, MA, USA), CD14-positive monocytes were sorted with a magnetic device. Informed consent was obtained from all patients before samples were collected, and all related procedures were approved by the internal review and ethics boards of the Renmin Hospital of Wuhan University. 

### 4.2. RNA Extraction, cDNA Synthesis, Genome DNA Isolation, and Quantitative Real-Time Polymerase Chain Reaction (qRT-PCR)

Total RNA extraction from cells/tissues was performed using Trizol reagent (TaKaRa Biotechnology, Dalian, China). Extracted total RNAs were measured by NanoDrop 2000 spectrophotometry (ThermoFisher Scientific, Waltham, MA, USA). For circRNA and mRNA, reverse transcriptions were performed using the PrimeScript RT Master Mix (TaKaRa Biotechnology, Dalian, China). For miRNA, reverse transcriptions were performed using the miRNA 1st Strand cDNA Synthesis Kit (Vazyme, Nanjing, China) with specific sperm-loop primers. According to the manufacturer’s instructions, genome DNA was isolated using a QIAamp DNA Mini Kit (QIAGEN, Germantown, MD, USA). qRT-PCR analyses were performed using the ChamQ SYBR qPCR Master Mix (Vazyme, Nanjing, China). qRT-PCR was performed using the QuantStudio 5 Real-Time PCR system (ThermoFisher Scientific, Waltham, MA, USA), and relative gene expression was calculated by the 2^−∆∆CT^ method. The relative expressions of circRNA and mRNA were normalized to GAPDH, and U6 for miRNAs, respectively. The primers for circRNAs and mRNAs are listed in Table 4, and the primers for miRNAs are listed in Table 5.

### 4.3. Library Construction and Sequencing

Total RNAs were isolated from decidual macrophages in eight pairs of RM patients and healthy subjects. The quality of RNA samples was assessed and used to construct a library. Using a Ribo-Zero RNA Removal Kit (Illumina, San Diego, CA, USA), rRNA was removed from total RNA, then fragmented to approximately 200 bp with RNaseR. Following purification of the RNA fragments, cDNA first-strand and second-strand synthesis was performed, followed by adaptor ligation and low-cycle enrichment according to the instructions of NEBNext^®^ UltraTM RNA Library Prep Kit for Illumina (NEB, Ipswich, MA, USA) and sequencing on the HiSeq 3000 using two 150 bp segments. We used EdgeR software (version 3.16.5) to normalize the data. To identify the differentially expressed circRNAs, |Log2Fold change| > 1 and *p* < 0.05 were selected as the cut-off criteria.

### 4.4. Functional Enrichment Analysis

The Gene Ontology (GO) annotation and Kyoto Encyclopedia of Genes and Genomes (KEGG) enrichment of the differentially expressed circRNAs were analyzed with the parental transcripts of these circRNAs. The GO categories were derived from Gene Ontology (http://www.geneontology.org (accessed on 25 March 2021)) and comprised biological process (BP), molecular function (MF), and cellular component (CC). The KEGG database (https://www.genome.jp/kegg (accessed on 25 March 2021)) is a widely used database storing information about genomes, biological pathways, diseases, and drugs. A *p*-value of <0.05 was used as the threshold to indicate a significant difference.

### 4.5. Agarose Gel Electrophoresis and Sanger Sequencing

PCR products were subjected to the 0.8% agarose gel electrophoresis method. The back-splicing sequence of Hsa_circ_0003241 was verified through the circBase database (http://www.circbase.org/ (accessed on 20 September 2021)). Amplification products of Hsa_circ_0003241 were confirmed by Sanger sequencing performed by Tsingke Biotech (Beijing, China).

### 4.6. Cell Line Culture and Macrophage Polarization

The human leukemia monocytic cells (THP-1) and the human embryonic kidney epithelial cell line (HEK-293T) were the kind gifts of Prof. Xi Zhou (State Key Laboratory of Virology, Wuhan Institute of Virology, Chinese Academy of Science, Wuhan, China). The THP-1 cells were cultured in Roswell Park Memorial Institute (RMPI)-1640 medium (Thermo, Waltham, MA, USA) supplemented with 10% (volume fraction) fetal bovine serum (FBS, Gibco, Waltham, MA, USA) and 1% penicillin–streptomycin solution (P/S, Gibco, Waltham, MA, USA) at 37 °C and 5% CO_2_. THP-1-derived macrophage (TDM) cell differentiation was induced by stimulation with Phorbol-12-Myristate-13-Acetate (PMA, Sigma-Aldrich, Zwijndrecht, The Netherlands) for 24 h at a concentration of 100 ng/mL. After cell attachment, TDM cells were stimulated with LPS (20 ng/mL, Sigma-Aldrich, St. Louis, MO, USA) and IFN-γ (20 ng/mL, Invitrogen, Waltham, MA, USA) for M1 polarization or IL-4 (20 ng/mL, Invitrogen, Waltham, MA, USA) and recombinant human IL-13 (20 ng/mL, Sigma-Aldrich, St. Louis, MO, USA) for M2 polarization. Moreover, the HEK-293T cells were propagated in Dulbecco’s modified Eagle’s medium (DMEM, Gibco, Waltham, MA, USA) supplemented with 10% FBS and 1% P/S at 37 °C and 5% CO_2_.

### 4.7. RNaseR and Actinomycin D (Act-D) Treatment

To detect the stability of circRNA, RNaseR and Act-D treatment assays were performed. RNaseR, a member of the RNA superfamily of exoribonucleases, is a 3′-5′ exoribonuclease that releases 5′ nucleoside monophosphates. Briefly, total RNA samples extracted from TDM cells were incubated with or without 2 U/ug RNaseR (Geneseed Biotech, Guangzhou, China) at 37 °C for 15 min. Then, RNA was purified using the RNeasy MinElute Cleanup kit (Qiagen, Hilden, Germany). Furthermore, the Act-D treatment assay quantified their half-lives. The TDM cells were exposed to Act-D (Sigma-Aldrich, St. Louis, MO, USA) for 0, 4, 8, 12, and 24 h at a concentration of 2 µg/mL against new RNA synthesis. Then, the relative expression levels of RNAs were measured using the qRT-PCR assay.

### 4.8. Nuclear and Cytoplasmic Separation

Nuclear and cytoplasmic separation was performed following the manufacturer’s protocol. In brief, RNA was isolated from nuclear and cytoplasmic fractions using a Paris kit (Invitrogen, Waltham, MA, USA). Subsequently, the relative expression of circKIAA0391 was explored by qRT-PCR. GAPDH was the cytoplasmic control, while U6 was the nuclear control.

### 4.9. Lentivirus Package and Cell Infection

The circKIAA0391 overexpression plasmid (OE-circKIAA0391) was generated by inserting the full length of the circKIAA0391 sequence into a lentiviral vector (Tsingke, Wuhan, China). Liposofectamine^TM^ 2000 (Invitrogen, Waltham, MA, USA) was used to co-transfect the overexpression plasmid with the psAX2 packaging plasmid and the pMD2.G envelope plasmid. A cellulose acetate filter (Millipore, Burlington, MA, USA) was used to filter the lentivirus supernatants collected 48 h post-transfection. Then, the THP-1 cells were infected by the recombinant lentivirus carrying circKIAA0391 overexpression plasmids or negative control plasmids for 48 h. The cells were subsequently selected using puromycin resistance for 1 week, and the surviving cells were regarded as stable circKIAA0391 overexpression cells or their matching control cells. Then, the relative expression of circKIAA0391 was examined by qRT-PCR.

### 4.10. Prediction of Target miRNA and miRNA Target Genes

miRanda (www.microrna.org/ (accessed on 12 December 2021)), RNAhybrid (https://bio.tools/rnahybrid (accessed on 12 December 2021)), and TargetScan (http://genes.mit.edu/targetscan/ (accessed on 12 December 2021)) databases were used to predict target miRNAs. The miRDB (mirdb.org/miRDB/ (accessed on 12 December 2021)), miRTarBase (mirtarbase.mbc.nctu.edu.tw (accessed on 12 December 2021)), and miRwalk (http://www.ma.uni-heidelberg.de/apps/zmf/miRwalk/ (accessed on 12 December 2021)) databases were also used.

### 4.11. Cell Transfection

siRNAs targeting the back-spliced sequence of circKIAA0391 were designed and synthesized by Ribobio Biotech (Guangzhou, China). To ensure the knockdown efficiency, two siRNA sequences were synthesized. Furthermore, miR-512-5p mimics (50 nM), miR-512-5p inhibitors (50 nM), and their matching negative controls were obtained from GenePharma Co. (Shanghai, China). Lipofectamine^TM^ 2000 reagent was used for cell transfections. After transfection, cells were collected for the subsequent assays. The detailed sequence of RNA oligos is listed in Table 6.

### 4.12. Protein Extraction and Immunoblotting

Cell lysates were prepared with cell lysis buffer (ThermoFisher Scientific, Waltham, MA, USA). The total protein was quantified with the BCA Protein Assay Kit (Beyotime, Haimen, China) and denatured at 95 °C for 10 min. Next, 8–12% sodium dodecyl sulfate-polyacrylamide gel electrophoresis (SDS-PAGE) was used to separate the protein, which was then transferred to polyvinylidene fluoride (PVDF) membranes. Then, incubation for 1 h at room temperature in Tris-buffered saline with Tween-20 (TBST) and 5% skim milk was performed to block the membrane. After that, the membrane was probed overnight at 4 °C using primary antibodies recognizing MITA (Abcam, Waltham, MA, USA, ab239074), PCNA (Abcam, Waltham, MA, USA, ab92552), BAX (1:1000, Abcam, Waltham, MA, USA, ab32503), Bcl-2 (Abcam, Waltham, MA, USA, ab182858), NLRP3 (Abcam, Waltham, MA, USA, ab263899), GSDMD (Abcam, Waltham, MA, USA, ab219800), cleaved N-terminal GSDMD (Abcam, Waltham, MA, USA, ab215203), caspase-1 (Abcam, Waltham, MA, USA, ab207802), HA tag (Abcam, Waltham, MA, USA, ab9110), DDDDK tag (Abcam, Waltham, MA, USA, ab205606), and GAPDH (1:2500, Abcam, Waltham, MA, USA, ab9485). After washing with TBST buffer three times, the membranes were incubated with horseradish peroxidase (HRP)-conjugated goat-anti-rabbit antibody (1:10,000, Abcam, Waltham, MA, USA) at room temperature for 1 h. Finally, after washing again with TBST solution five times, the bands were detected by ECL (Beyotime Biotechnology).

### 4.13. Dual-Luciferase Reporter Assay

The potential binding sites between miR-512-5p and circKIAA0391 were predicted using the TargetScan database. The wild-type and mutated sequences of circKIAA0391 (circKIAA0391-WT and circKIAA0391-Mut) were inserted in the pmiR-RB-ReportTM plasmid (Ribobio, Guangzhou, China). We transfected TDM cells with luciferase reporter plasmids and miR-512-5p mimics or the negative control (NC) for 24 h in 24-well plates. Following the manufacturer’s instructions, the dual-luciferase reporter assay system (Promega, Madison, WI, USA) was used to measure the luciferase activity. Renilla luciferase activity was used as a control for normalizing the results.

### 4.14. RNA Pull-Down Assay

Sense- and antisense-circKIAA0391 probes were synthesized and labeled with biotin by Ribobio Co. Ltd. (Guangzhou, China). The primers for biotin-labeled circKIAA0391 probes are listed in Table 7. Biotinylated miR-512-5p and biotinylated NC probes were synthesized by GenePharma (Shanghai, China). The miRNA probes are listed in Table 8. In brief, TDM cells were collected, and whole-cell lysates were extracted, followed by incubation with each probe at 4 °C for 4 h. Then, streptavidin-coupled agarose beads were added to the cell lysates for another 2 h. After washing, the biotin-coupled RNA complex was pulled down and extracted. Then, the abundances of circKIAA0391, miR-512-5p, and predicted target mRNAs were detected by qRT-PCR.

### 4.15. Immunoprecipitation (IP), Co-Immunoprecipitation (CO-IP), and RNA Immunoprecipitation

HEK-293T cells were transiently co-transfected for co-IPs with HA-MITA and Flag-GSDMD or HA-MITA and Flag-NLRP3. Forty-eight hours later, cells were harvested, and the protein amounts were determined using the BCA assay. Cell extracts were incubated for 12–16 h with the indicated antibodies, followed by additional incubation with protein A/G beads (Invitrogen, Waltham, MA, USA) for 2–4 h. For endogenous co-IP, antibodies were conjugated to resin using the CO-IP kit (Pierce, Appleton, WI, USA). The experiments were carried out in the presence of the proteasome inhibitor MG132 (20 μM). Input and eluted proteins from the pull-down and co-immunoprecipitation assays were separated by SDS-PAGE and probed with the corresponding antibodies in Western blot analyses. The RIP experiments were conducted using the Magna RIP RNA-Binding Protein Immunoprecipitation Kit (Millipore, Bedford, MA, USA), following the manufacturer’s instructions. In brief, TDM cells were transfected with miR-512-5p mimics or miR-NC. Cells were harvested after transfection for 48 h and lysed in RIPA buffer (Cell Signaling Technology, Danvers, MA, USA) with protease inhibitor cocktail (Invitrogen, Waltham, MA, USA) and RNasin^®^ Plus Ribonuclease Inhibitor (Promega, Madison, WI, USA). Before IP, a positive control in the RIP assay, called “input”, was obtained from the cell lysate. Then, the remaining lysate was cultivated with magnetic beads, coupled with the AGO2 antibody (Abcam, Waltham, MA, USA, ab186733) or negative control IgG antibody (Proteintech, B900620, Wuhan, China), with rotation overnight at 4 °C. Finally, the RNA was purified from the RNA–protein complexes bound to the beads, and then the circKIAA0391 and miR-512-5p were determined using qRT-PCR.

### 4.16. Flow Cytometry (FCM)

For flow cytometry and immunofluorescence staining, CD206 was initially selected as a marker of M2, but our preliminary study found a low positive rate for CD206 in flow cytometry, leading us to hypothesize that CD206 would be required to break the membrane of the cells when performing the assay. Thus, the surface antigen CD209 was selected as a marker for M2 cells since another part of our study required multiple M2 cells for flow cytometric sorting. Collectively, to detect the polarization status of macrophages, we used qRT-PCR and flow cytometry. The mRNA level of iNOS and TNF-α and the surface marker of CD86 were used as the marker of M1. Furthermore, the mRNA level of ARG-1 and IL-10 and the surface marker of CD209 were used as the marker of M2. We still used CD206 as a marker for immunofluorescence since this experiment was performed at the tissue level. Using qRT-PCR and flow cytometry, we determined the polarization of macrophages and the combination of both data made the results more convincing.

Macrophage phenotypes were analyzed via the surface expression of specific surface markers. In the case of patients who underwent an abortion, the decidual tissue was collected and washed with PBS to remove any residual blood left behind. The tissue specimens were transferred to an ultra-clean bench at 4 °C to be processed. As a first step, the tissues were cut into small pieces with surgical scissors and ground to prepare a suspension of the cells. After centrifuging the cells and removing residual erythrocytes using an erythrocyte lysis solution, the lymphocytes were separated with a lymphocyte isolation solution. We harvested and washed the cells with ice-cold PBS and adjusted the cell concentration to 10^6^ cells/mL. The cells were then immunolabeled with APC-CY7-conjugated Fixable Viability Stain780 (Becton, Dickinson and Company, Franklin Lakes, NJ, USA), APC-conjugated anti-human CD86 antibodies (Biolegend, San Diego, CA, USA) or Percp cy5.5-conjugated anti-human CD209 antibodies (Biolegend, San Diego, CA, USA). Then, the cells were incubated with the appropriate conjugated primary antibody for 30 min at 4 °C in the dark. The cells were centrifuged at 500 g/min and resuspended in PBS solution. Cells were then filtered through nylon mesh filters and washed with PBS. All of the data were acquired through flow cytometry (FACS Canto II; Becton, Dickinson and Company, Franklin Lakes, NJ, USA) and processed using the FlowJo software 10.8.1 (Tree Star, Ashland, OR, USA).

### 4.17. Immunohistochemistry (IHC)

For the preparation of paraffin-embedded sections, fresh decidual tissues from RM and HC patients were fixed in 10% formalin for 24 h at room temperature, washed twice with PBS, and then stored in 70% ethanol solution at 4 °C until they were embedded in paraffin and sectioned at 4 μm. In each group, immunohistochemistry for MITA was performed. The paraffin-embedded sections were heated for 30 min at 65 °C, deparaffinized in xylene, and rehydrated through graded ethanol at room temperature. A 0.05 M Tris HCl buffer (pH 7.6) was used to prepare solutions and for washes between various steps. Incubations were performed in a humidified chamber. Sections were treated for 40 min at room temperature with 2% BSA and incubated overnight at 4 °C with primary antibodies. Horseradish peroxidase activity was visualized by treatment with H_2_O_2_ and 3,3′-diaminobenzidine for 5 min.

### 4.18. Multiplexed Immunofluorescence (mIF)

A Bond RX automated stainer (Leica Biosystems, Buffalo Grove, IL) was used to stain 4 mm thick formalin-fixed, paraffin-embedded decidual tissues. After incubating the cells for an hour in 5% BSA (0.1% Triton X-100), nonspecific binding was blocked. Anti-CD86 antibodies (Abcam, Waltham, MA, USA, ab239075), CD206 antibodies (Abcam, Waltham, MA, USA, ab64693), and MITA antibodies were incubated on the slides. Afterward, they were incubated with Cy3, fluorescein isothiocyanate (FITC), or Cy5. The nuclei of the cells were stained using DAPI. The images were observed by using a confocal microscope.

### 4.19. Statistical Analysis

Analysis of the collection and statistical data were processed with the SPSS software version 22.0 (SPSS, Chicago, IL, USA) using Student’s *t*-test. All of the experiments were repeated at least three times for each group. Correlations were performed by Pearson correlation analysis. Data were expressed as the means ± standard deviation and were plotted using the GraphPad Prism program version 9.4.0 (GraphPad Prism8; GraphPad, San Diego CA, USA). Two-sided *p* < 0.05 was considered to represent a statistically significant difference.

## 5. Conclusions

In summary, the present study showed that MITA is highly expressed in decidual macrophages and may be related to the pathology of RM. Further mechanical results showed that circKIAA0391 promotes macrophage apoptosis and proinflammatory polarization in TDM cells partly by depending on the miR-512-5p/MITA network. Therefore, our findings not only help to understand the pathogenesis of RM but also indicate that the circKIAA0391/miR-512-5p/MITA axis may be a potential target for RM treatment.

## Figures and Tables

**Table 1:** The basal characteristics of the 15 up- and downregulated circRNAs.

circName	circBase	Gene Symbol	Regulation	Chromosome	Strand	log2 Fold Change	*p*-Value
hsa_circ:chr19:23556544-23557566	NA	*ZNF91*	Up	19		7.326852132	0.001008545
hsa_circ:chr19:45766371-45769539	hsa_circ_0051449	*MARK4*	Up	19	+	6.21219094	0.00419019
hsa_circ:chr11:2997254-3000467	hsa_circ_0003834	*NAP1L4*	Up	11	-	6.126442056	0.001458512
hsa_circ:chr12:79370373-79430353	NA	*ENSG00000257191.1*	Up	12		6.081986147	0.001452721
hsa_circ:chr20:3925824-3955047	hsa_circ_0005736	*RNF24*	Up	20	-	6.037103066	0.005254964
hsa_circ:chr3:57875768-57882659	hsa_circ_0002693	*SLMAP*	Up	3	+	6.003651004	0.015337453
hsa_circ:chr12:46339032-46345510	hsa_circ_0025971	*SCAF11*	Up	12	-	5.853058519	0.007173809
hsa_circ:chr12:66603236-66611015	hsa_circ_0003753	*IRAK3*	Up	12	+	5.837553392	0.002738124
hsa_circ:chr19:23316882-23318845	hsa_circ_0008122	*ZNF730*	Up	19	+	5.734581936	0.007734256
hsa_circ:chr8:125332327-125343033	hsa_circ_0085496	*TMEM65*	Up	8	-	5.658587239	0.005583935
hsa_circ:chr11:76670023-76709865	hsa_circ_0003320	*ACER3*, *ENSG00000254988.1*	Up	11	+	5.599083086	0.02172101
hsa_circ:chr2:48896861-48898819	NA	*GTF2A1L*, *STON1-GTF2A1L*	Up	2		5.578529864	0.001355323
hsa_circ:chr14:35595941-35596817	hsa_circ_0003241	*KIAA0391*	Up	14	+	5.55641131	0.000138713
hsa_circ:chr5:50045985-50059076	hsa_circ_0005809	*PARP8*	Up	5	+	5.483037782	0.026741084
hsa_circ:chr14:33046326-33069998	NA	*AKAP6*	Up	14		5.286474623	0.000989938
hsa_circ:chr9:33960824-33973235	hsa_circ_0001850	*UBAP2*	Down	9	-	−9.279337894	3.75 × 10^−7^
hsa_circ:chr16:31733947-31734674	hsa_circ_0007059	*ZNF720*	Down	16	+	−8.284332147	0.000951794
hsa_circ:chr1:51926763-51930998	hsa_circ_0007330	*EPS15*	Down	1	-	−7.572275262	0.001726882
hsa_circ:chr17:80858527-80869665	hsa_circ_0005245	*TBCD*	Down	17	+	−6.984132264	0.002847385
hsa_circ:chr2:120830754-120836157	hsa_circ_0001064	*EPB41L5*	Down	2	+	−6.824457064	0.003379807
hsa_circ:chr15:42553156-42560230	hsa_circ_0034803	*TMEM87A*	Down	15	-	−6.792628973	0.003543211
hsa_circ:chr19:20828490-20829211	hsa_circ_0050277	*ENSG00000269110.1*, *ZNF626*	Down	19	-	−6.603963846	0.004149898
hsa_circ:chr2:44931422-44942492	hsa_circ_0006530	*CAMKMT*	Down	2	+	−6.555402687	0.004378012
hsa_circ:chr12:129429871-129442188	hsa_circ_0029410	*GLT1D1*	Down	12	+	−6.344570125	0.002066197
hsa_circ:chr9:107513237-107521452	NA	*NIPSNAP3A*	Down	9		−6.33989564	0.006185369
hsa_circ:chr19:8612920-8613201	NA	*MYO1F*	Down	19		−6.267903258	0.000569616
hsa_circ:chr7:77210744-77214898	NA	*PTPN12*	Down	7		−6.022293846	0.008698481
hsa_circ:chr7:107595912-107601094	hsa_circ_0081931	*LAMB1*	Down	7	-	−5.993055482	7.32 × 10^−8^
hsa_circ:chr14:67812485-67814170	NA	*ATP6V1D*	Down	14		−5.933284827	0.003570891
hsa_circ:chr10:116719485-116734144	hsa_circ_0020091	*TRUB1*	Down	10	+	−5.861799423	0.011216616

**Table 2:** The target miRNAs of circKIAA0391 predicted by miRanda software.

miRNA	Gene	Max Score	Max Energy	Positions
hsa-miR-1306-5p	hsa_circ_0003241	145	−25.1	135
hsa-miR-181b-5p	hsa_circ_0003241	153	−14.53	93
hsa-miR-193b-3p	hsa_circ_0003241	141	−15.2	35
hsa-miR-2355-3p	hsa_circ_0003241	159	−14.87	8
hsa-miR-23a-3p	hsa_circ_0003241	143	−16.02	124
hsa-miR-23c	hsa_circ_0003241	146	−17.17	123
hsa-miR-3190-5p	hsa_circ_0003241	142	−15.1	38
hsa-miR-3692-3p	hsa_circ_0003241	159	−21.94	46
hsa-miR-378a-3p	hsa_circ_0003241	150	−17.87	82
hsa-miR-378b	hsa_circ_0003241	148	−17.17	84
hsa-miR-378c	hsa_circ_0003241	157	−21.41	79
hsa-miR-378d	hsa_circ_0003241	147	−16.43	83
hsa-miR-378e	hsa_circ_0003241	147	−15.61	84
hsa-miR-378f	hsa_circ_0003241	147	−15.61	83
hsa-miR-378h	hsa_circ_0003241	149	−19.13	80
hsa-miR-378i	hsa_circ_0003241	150	−17.25	80
hsa-miR-3940-3p	hsa_circ_0003241	146	−27.13	46
hsa-miR-422a	hsa_circ_0003241	154	−19.52	82
hsa-miR-4427	hsa_circ_0003241	163	−18.78	72
hsa-miR-4446-5p	hsa_circ_0003241	150	−13.42	103
hsa-miR-4659b-3p	hsa_circ_0003241	154	−16.73	87
hsa-miR-4755-5p	hsa_circ_0003241	152	−14.07	105
hsa-miR-512-5p	hsa_circ_0003241	154	−17.02	78
hsa-miR-5585-3p	hsa_circ_0003241	141	−14.58	69
hsa-miR-5587-5p	hsa_circ_0003241	161	−25.31	143
hsa-miR-5706	hsa_circ_0003241	143	−20.49	84
hsa-miR-584-5p	hsa_circ_0003241	151	−18.58	58
hsa-miR-588	hsa_circ_0003241	140	−19.8	35
hsa-miR-6757-3p	hsa_circ_0003241	154	−18.93	38
hsa-miR-6787-3p	hsa_circ_0003241	153	−18.35	75
hsa-miR-6830-3p	hsa_circ_0003241	150	−18.73	147
hsa-miR-6856-3p	hsa_circ_0003241	147	−22.82	48,120
hsa-miR-892b	hsa_circ_0003241	150	−22.82	36

**Table 3:** Clinical characteristics of the women included in this study.

	Microarray Cohort			Validation Cohort		
	RM	HC	*p*-Value	RM	HC	*p*-Value
Age (years)	29.13 ± 3.76	30.63 ± 4.53	0.483	31.92 ± 5.03	30.68 ± 4.23	0.35
Gestation durations (days)	78.00 ± 15.78	75.38 ± 11.30	0.708	78.56 ± 13.58	77.52 ± 12.90	0.783

**Table 4:** Primers for circRNAs and mRNAs.

Gene Symbol	Primer	Sequences (5′-3′)
hsa_circ_0001850	F	AGAAGACTGGACTGAAGATGATTTG
	R	GTTCAGTTCCCTCATCTGCACC
Hsa_circ_0003241	F	ACAGAAAGACAACACCTCAGTGTTC
	R	ATCCCTCATGATTTTTCCCTTAAG
hsa_circ_0006552	F	GGAGATGTCTCTTTGCAAGAAAGT
	R	CAAATGTCAGGGACTGCTTGG
hsa_circ_0008641	F	GGTGACTTTGTGTGCCCCATG
	R	GCGTTGGAAGAGAGCTGGCTAC
hsa_circ_0081931	F	CAGCTTGCCTGTGTTTGTGATC
	R	CTGCAAACATCTGTCATCGGTG
ARG-1	F	CATCCCTAATGACAGTCCCTTT
	R	CAGGAGGAAAGATACAGGTTGT
ARIH1	F	AGAGAATGCCACAGAGGTGC
	R	CCCTTCGTCGACTCTCACAG
BAZ2A	F	ACTGTATCTCACACTACTAC
	R	GAAGGTTAGTGTTATGACTT
BCL2L2	F	CAAGGAGATGGAACCACTGGTG
	R	CCGTATAGAGCTGTGAACTCCG
CTNNB1	F	CACAAGCAGAGTGCTGAAGGTG
	R	GATTCCTGAGAGTCCAAAGACAG
FAM83C	F	CCTGGAGAAGTTCGTCCTCATTG
	R	GTCAAAGTCTTCCACGATGCGG
GPRC5B	F	ACAATGCAGCTCTCCGAACAG
	R	TGATACACGTTGCTTCTAAACGG
IL-10	F	GTTGTTAAAGGAGTCCTTGCTG
	R	TTCACAGGGAAGAAATCGATGA
iNOS	F	TGCCACGGACGAGACGGATAG
	R	CTCTTCAAGCACCTCCAGGAACG
KIAA0391	F	GGAAGCACATGCTAAGACGGAG
	R	GTGCAGTGTGGCATACAGAAGG
MITA	F	GGTGCCTGATAACCTGAGTATGG
	R	GTTGCTGTAAACCCGATCCTTG
P21	F	CTACTGAGGAGCCAGCGTCTA
	R	CTGCCCATCATCATGACCT
PTMA	F	GGCTGACAATGAGGTAGACGAAG
	R	GTAGCTGACTCAGCTTCCTCATC
TNF-α	F	ATGTCTCAGCCTCTTCTCATTC
	R	GCTTGTCACTCGAATTTTGAGA
VHL	F	CGGGATCCATGCCCCGGAGGGCGGAGAA
	R	CCGCTCGAGTCAATCTCCCATCCGTTGATGTG
GAPDH	F	GTCTCCTCTGACTTCAACAGCG
	F	ACCACCCTGTTGCTGTAGCCAA

**Table 5:** Primers for miRNAs.

Gene Symbol	Primer	Sequences (5′-3′)
miR-4427	Reverse	GTCGTATCCAGTGCAGGGTCCGAGGTATTCGCACTGGATACGACACTCTT
	F	CGCGCGTCTGAATAGAGTCTG
	R	AGTGCAGGGTCCGAGGTATT
miR-512-5p	Reverse	GTCGTATCCAGTGCAGGGTCCGAGGTATTCGCACTGGATACGACGAAAGT
	F	GCACTCAGCCTTGAGGGC
	R	AGTGCAGGGTCCGAGGTATT
miR-5706	Reverse	GTCGTATCCAGTGCAGGGTCCGAGGTATTCGCACTGGATACGACAGCTTC
	F	CGCGTTCTGGATAACATGCT
	R	AGTGCAGGGTCCGAGGTATT
miR-6757-3p	Reverse	GTCGTATCCAGTGCAGGGTCCGAGGTATTCGCACTGGATACGACTGGGGA
	F	GCGAACACTGGCCTTGCTA
	R	AGTGCAGGGTCCGAGGTATT
miR-6787-3p	Reverse	GTCGTATCCAGTGCAGGGTCCGAGGTATTCGCACTGGATACGACCTGGAG
	F	CGTCTCAGCTGCTGCCCT
	R	AGTGCAGGGTCCGAGGTATT
U6	Reverse	GTCGTATCCAGTGCAGGGTCCGAGGTATTCGCACTGGATACGACAAAATATGG
	F	GCTCGCTTCGGCAGCACATATAC
	R	AGTGCAGGGTCCGAGGTATT

**Table 6:** RNA oligos used in this study.

Symbol	Sense (5′-3′)
si-circKIAA0391-1	ACAACACCUCAGUGUUCCUTT
si-circKIAA0391-2	ACACCUCAGUGUUCCUGGATT
si-MITA-1	CATGGTCATATTACATCGGAT
si-MITA-2	GCTGGCATGGTCATATTACAT
Negative Control	UUCUCCGAACGUGUCACGUTT

**Table 7:** Primers for biotin-labeled circRNA pull-down assay.

Symbol	F/R	Sense (5′-3′)
Sense (5′-3′)	F	TAATACGACTCACTATAGGGTGTTCCTGGAAAACAATGGAAAGGACAATTCACCACAGTC
	R	CTGAGGTGTTGTCTTTCTGTACTGGTCACCTCCATCTATC
Antisense (5′-3′)	F	TAATACGACTCACTATAGGGCTGAGGTGTTGTCTTTCTGTACTGGTCACCTCCATCTATC
	R	TGTTCCTGGAAAACAATGGAAAGGACAATTCACCACAGTC

**Table 8:** The biotin-labeled miRNA probes for miRNA pull-down assay.

Symbol	Sense (5′-3′)
biotin-miR-512-5p probes	CAC+UCAGCCU+UGAGGGCAC+UUUC
biotin-miR-512-5p NC probes	AAAAAAAAAAAAAAAAAAAAAAAAA

## Data Availability

All other data is available from the author upon reasonable request.

## Supplementary Materials

The following supporting information can be downloaded at: https://www.mdpi.com/article/10.3390/ijms24119545/s1.
