# Supplementary material for: MITA Promotes Macrophage Proinflammatory Polarization and Its circRNA-Related Regulatory Mechanism in Recurrent Miscarriage
Source: Int J Mol Sci. 2023 May 31;24(11):9545. doi: 10.3390/ijms24119545 (PMC10253871; doi:10.3390/ijms24119545)
Supplement: Supplementary file 1 [file ijms-24-09545-s001.zip › ijms-2301612-supplementary.docx]

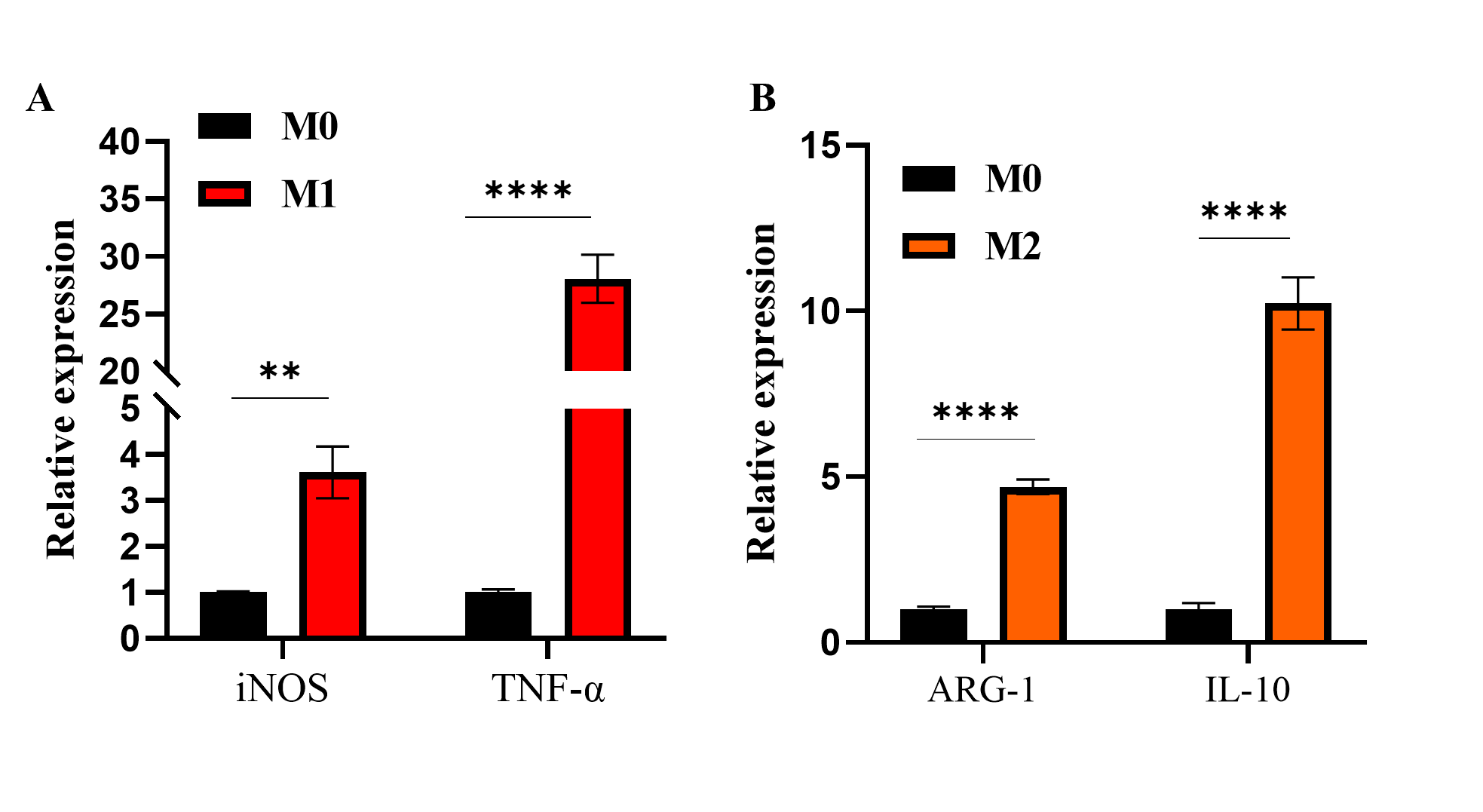
**Supplementary Figure S1.** Validation of M1 and M2 cells. **A**)The mRNA level of M1 markers by qRT-PCR. **B**)The mRNA level of M2 markers by qRT-PCR. Statistical comparison between two groups was made by unpaired *t*-test. ** *p <* 0.01; **** *p <* 0.0001.


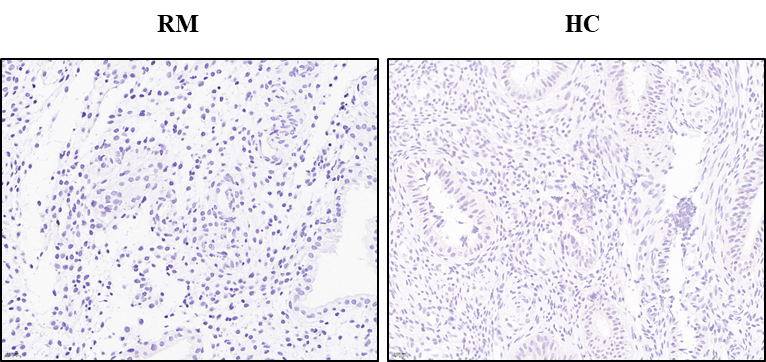


**Supplementary Figure S2:** The negative control for IHC of the decidual tissues from RM and HC patients. 40×, Scale bar = 0.020 mm.


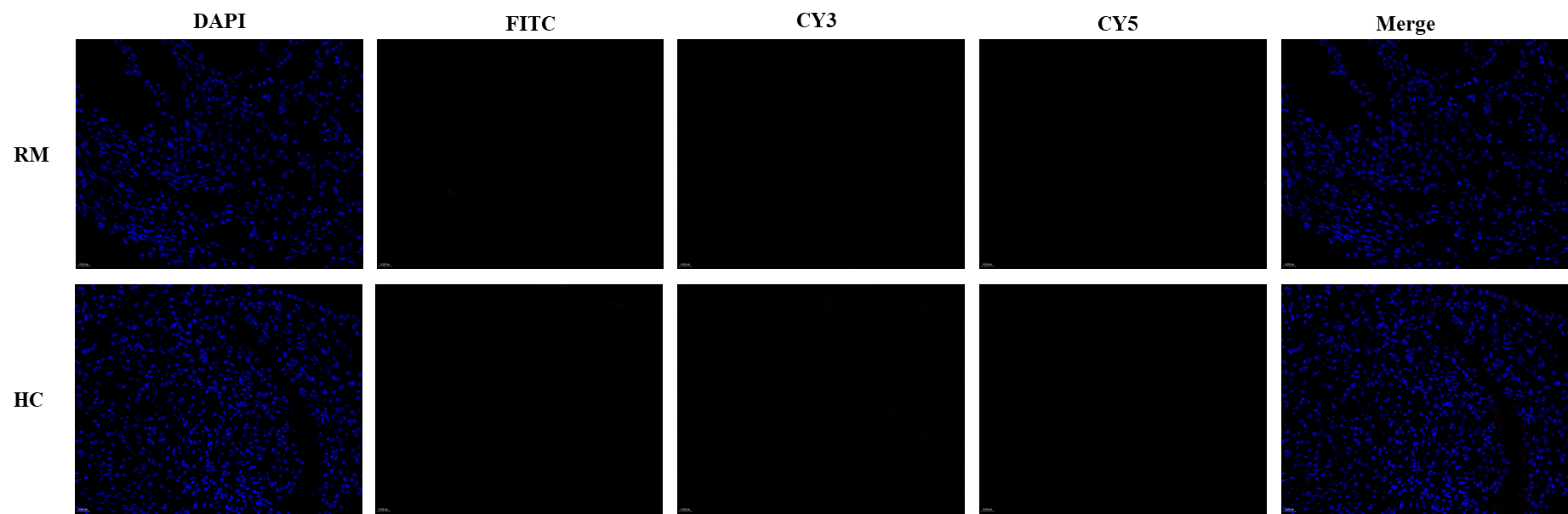


**Supplementary Figure S3:** The negative control for mIF staining of the decidual tissues from RM and HC patients. 40×, Scale bar = 0.020 mm.
